# Callus Formation and Mineralization after Fracture with Different Fixation Techniques: Minimally Invasive Plate Osteosynthesis versus Open Reduction Internal Fixation

**DOI:** 10.1371/journal.pone.0140037

**Published:** 2015-10-07

**Authors:** Haitao Xu, Zichao Xue, Haoliang Ding, Hui Qin, Zhiquan An

**Affiliations:** Department of Orthopedics, Shanghai Jiao Tong University Affiliated Sixth People’s Hospital, Shanghai, China; University of Ulm, GERMANY

## Abstract

Minimally invasive plate osteosynthesis(MIPO) has been considered as an alternative for fracture treatment. Previous study has demonstrated that MIPO technique has the advantage of less soft tissue injury compared with open reduction internal fixation (ORIF). However, the comparison of callus formation and mineralization between two plate osteosynthesis methods remains unknown. In this experiment, ulna fracture model was established in 42 beagle dogs. The fractures underwent reduction and internal fixation with MIPO or ORIF. Sequential fluorescent labeling and radiographs were applied to determine new callus formation and mineralization in two groups after operation. At 4, 8 and 12 weeks postoperatively, the animals were selected to be sacrificed and the ulna specimens were analyzed by Micro-CT. The sections were also treated with Masson staining for histological evaluation. More callus formation was observed in MIPO group in early stage of fracture healing. The fracture union rate has no significant difference between two groups. The results indicate that excessive soft tissue stripping may impact early callus formation. As MIPO technique can effectively reduce soft tissue injury with little incision, it is considered to be a promising alternative for fracture fixation.

## Introduction

There are a variety of options when operative treatment for fractures is required, among which, open reduction internal fixationis the most commonly used method and has obtained good outcomes, with the advantage of anatomical reduction[1–4]. However, this technique involves extensive soft tissue stripping and muscle retraction for adequate exposure, which leads to further devascularization of fracture fragments and disruption of periosteal blood supply. The risks of non-union and deep infection increase as a consequence[5–8]. Recently, many studies have reported the superiority of minimally invasive plate osteosynthesis (MIPO)[9–13]. Cadaveric vascular injection study for proximal humerus demonstrated that, the filling of the anterior and posterior vessels supplying the humeral head were undisturbed after the use of MIPO and locking plate[14]. MIPO technique is a safe and effective method with the advantages of less soft tissue injury, blood loss and decreased postoperative pain. Despite these merits, it is still in controversy for that which is the best method for plate osteosynthesis. Various clinical studies have been performed to compare the two techniques in union rate, union time and functional results. Moreover, there are studies showing that impaired blood supply could impact the formation and remodeling of callus, and that enhanced vascularization can promote callus formation[15, 16]. However, there are no studies known to us exploring the effect on callus formation and mineralization with two plate osteosynthesis methods. Our aim was to study the process of callus formation in animal model with different osteosynthesis techniques. This experiment would offer a new perspective for the comparison of MIPO and ORIF.

## Materials and Methods

### 2.1 Ethics statements

This study has been approved by the Animal Care and Use Committee of Shanghai Jiao Tong University Affiliated Sixth People’s Hospital.

### 2.2 Animal condition and grouping

The beagles were supplied by Agricultural college of Shanghai Jiao Tong University. Each animal was kept in one cage with sufficient food and water. 42 male beagle dogs (aged 2 years old, an average weight of 16 kg) were divided into two groups in this study. The fractures were made at the middle of ulna by a swing saw. Group A was treated with MIPO and synthesis locking compression plate (LCP), and Group B with ORIF and LCP. 7 dogs were randomly selected in each group to be sacrificed with 10% potassium chlorideat 4, 8 and 12 weeks postoperatively.

### 2.3 Surgical procedure

All the surgical procedures were performed under general anesthesia [3% pentobarbital sodium(1 mL/kg)] with administration of broad-spectrum antibiotic prophylaxis [ampicillin sodium (20 mg/kg)]. The operations were performed with the animal positioned in right lateral decubitus.

To make the ulna fracture model, a small incision (approximately 1.5 cm) was made at the middle of the dorsal part of forearm. Osteotomy was performed by swing saw. In the next step, Group A was treated with MIPO technique. Briefly, a distal and a proximal incision (approximately 2 cm for both incisions) were made approximately 1.5 cm lateral from the fracture site respectively. The LCP was inserted from the distal incision. In group B, an incision (approximately 12 cm) was made corresponding to the small incision made before over the fracture site to make only one incision in total. Periosteum was stripped for adequate exposure.The LCP fixation was performed after the fracture reduction. In two groups, three screws were utilized in both distal and proximal fragments respectively in an eight-hole LCP to obtain better fixation strength.

Anti-inflammatory drugs [ampicillin sodium (20 mg/kg)] were used for 5 days postoperatively.

### 2.4 Sequential fluorescent labeling

Fluorescent labeling was used after the operation to observe bone mineralization and deposition at different periods. For animals sacrificed at 8 weeks postoperatively, the dogs were subcutaneously administered with 20mg/kg calcein (CA, Sigma, USA), 30mg/kg alizarin red (AL, Sigma, USA) and 25mg/kg tetracycline (TE, Sigma, USA) at 2, 4 and 6 weeks after the operation, respectively. For animals sacrificed at 12 weeks postoperatively, the dogs were subcutaneously administered with 20mg/kg calcein (CA, Sigma, USA), 30mg/kg alizarin red (AL, Sigma, USA) and 25mg/kg tetracycline (TE, Sigma, USA) at 8, 9 and 10 weeks after the operation, respectively.

### 2.5 Radiological observation

To follow up the fracture union as well as new bone formation and mineralization, X-ray images for forearm were taken with animals under general anesthesia at 1 day, 4, 6 and 10 weeks postoperatively. The fracture union was defined as the presence of bridged callus in at least 3 of 4 cortices on two orthogonal radiographic views[17].

### 2.6 Evaluation of Micro-CT

Bone and mineralized callus structure and density were evaluated by Micro-CT. A SkyScan 1176 compact X-ray MicroCT scanner (Bruker, Belgium) was used for all scans with the beam set at 90 kV and 270μA. Scans were reconstructed at 18μm isotropic resolution. All samples were scanned within 3 days after sacrifice. All reconstructions were performed using Skyscan NRecon Program (Bruker, Belgium). Measurements and analysis were conducted using SkyScan Dataviewer (Bruker, Belgium) and SkyScan CTan (Bruker, Belgium).

Mineralized tissues were classified into either mineralized bone or callus depending on the density of the intact cortical bone in the ulna. Based on previously published researches, the density of callus considered to be mineralized was 35%–70% of the maximum density of the intact cortical bone[18]. The density of intact mineralized bone was determined to be over 70% of the maximum intensity of the cortical bone. Cylindrical volume of interest (VOI) with a length of 10mm was used to analyzed the fracture zone, centered at the midpoint of the fracture in longitudinal view.

Volume and density were calculated for mineralized bone and mineralized callus. Morphometric parameters measured were as follows: mineralized callus volume, mineralized callus density, bone volume and bone mineral density.

### 2.7 Sample preparation and histomorphometric observation

The ulna were harvested after the sacrifice of dogs at specific time point. Then the samples were fixed in buffered formalin (10%, pH7.4). The specimens were dehydrated in alcohols with ascending concentrations from 70% to 100%, and then embedded in polymethyl methacrylate. The specimens were cut in 8μm thick sections with a microtome (Leica, Hamburg, Germany).

The sections were observed under confocal laser scanning microscope for labeled fluorescent (Zeiss LSM710, Germany). Excitation and Emission Wavelengths of chelating fluorochromes were used as following: 488/517 nm (calcein, green), 543/617 nm (alizarin red, red), 405/615 nm (tetracycline, yellow), respectively. Three randomly selected sections collected from each sample were analyzed. Five photographs from the same area were taken: three fluorescence microscopy images including the fluorochromes calcein, alizarin red and tetracycline,one merged image of the three fluorescent labeling, and one image merged by an image taken from transmission light microscopy and the former merged image. The percentage of newly formed bone area was calculated at low magnification (10×).

The microscope images were digitally stored and evaluated histomorphometrically using a picture-analysis software system (Image-Pro Plus, Media Cybernetics, Rockville, MD, USA). In this software system, the number of pixels labeled for each fluorochrome in each image was measured as a percentage of the total mineralization area. This was done separately for green (calcein), red (alizarin red) and yellow (tetracycline). The data of fluorescent labeling represent the bone mineralization and regeneration at 2, 4, 6, 8, 9 and 10 weeks post-operation in dogs of different sacrificing time point.

The specimens were then further treated with Masson staining to observe the formation and mineralization of callus histologically. The sections were analyzed for the gap bridging in fracture area.

### 2.8 Statistical analysis

Data were all presented as mean ± standard deviation (Mean ± SD). The differences in bone area newly formed were measured using two-way ANOVAs. A *p*-value of 0.05 or less was considered asstatistically significant differences between the two groups. All statistical analysis was performed using an SAS 8.02 statistical software package (Cary, NC, USA).

## Results

### 3.1 General and radiographic observations

No intraoperative complications occurred in either group. All animals recovered well after surgery with no sign of infection or other complications.

**Fig 1:**
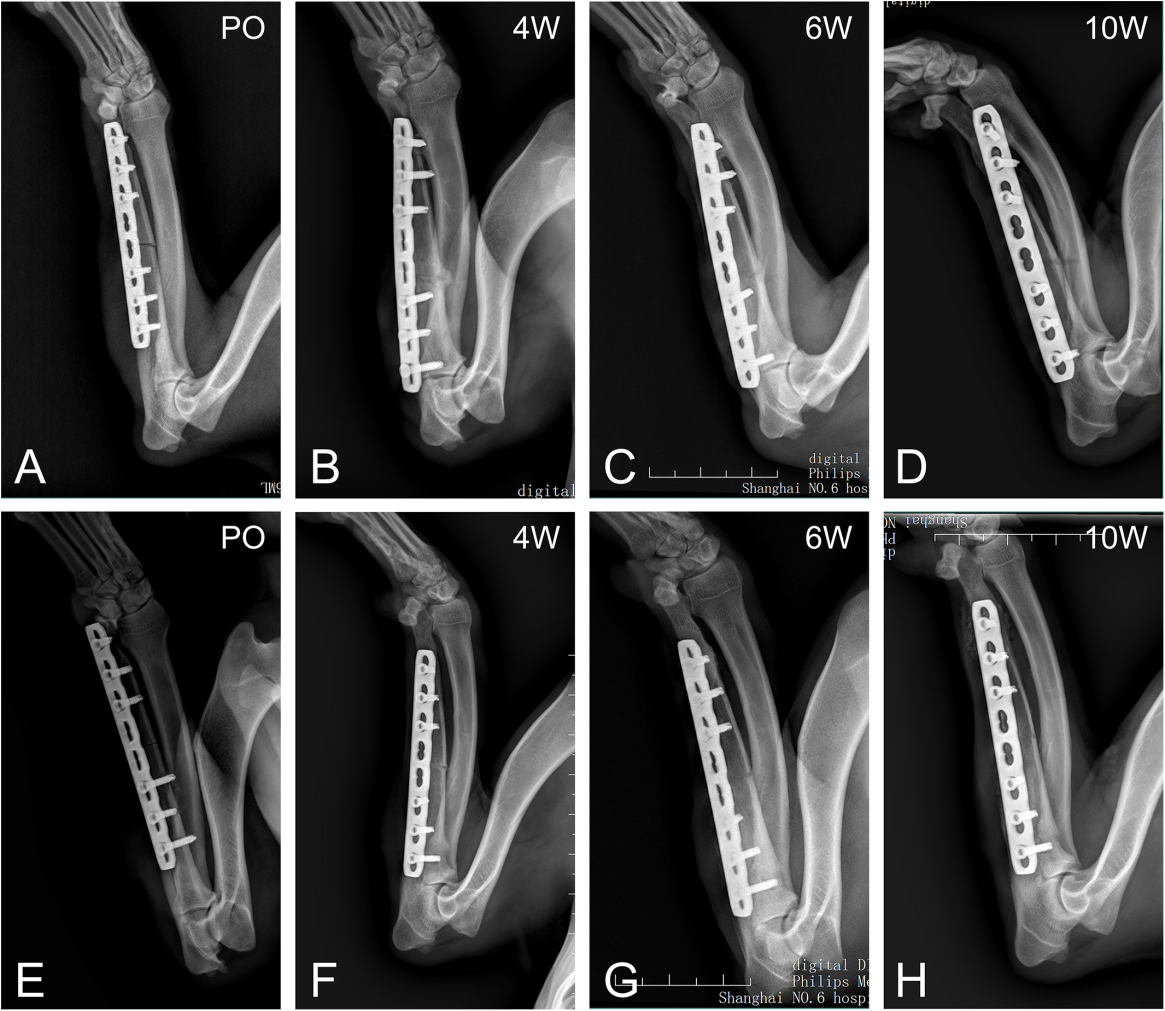
1 day, 4, 6, 10 weeks postoperative radiographs images for beagles treated with minimally invasive plate osteosynthesis (MIPO) (A-D) and open reduction internal fixation (ORIF) (E-H).

The fracture union was evaluated by radiographic image (Fig 1). There were 7 of 21 fractures obtaining union by 4 weeks in MIPO group, while there are 8 in ORIF group. At 6 weeks postoperatively, 11 of 14 animals in Group A obtained union and all fractures healed at 10 weeks postoperatively. In contrast, 12 of 14 dogs in Group B got fracture union at 6 weeks after the surgery, and all animals healed at 10 weeks postoperatively. MIPO group had a significantly larger fracture gap than ORIF group. The mediolateral translation in MIPO group was significantly greater than in ORIF group (Table 1).

**Table 1:** Mean ± SD for outcome measurements (fracture reduction and implant associated parameters for dogs in MIPO and ORIF groups). The P values indicate significant differences determined by unpaired t-test.

Outcome measures	MIPO group	ORIF group	P value
Time of surgery (minutes)	28.8±6.3	26.7±7.3	0.323
Fracture gap (mm)	0.5±0.3	0.3±0.2	0.017
Mediolateral translation (mm)	1.1±1.0	0.6±0.4	0.044
Varus-valgus angulation (degree)	3.4±1.8	2.6±2.1	0.182

### 3.2 Evaluation of Micro-CT

Micro-CT was used to calculate the volume and density of mineralized callus and bone. During the process of the experiment, mineralized callus formation at 4, 8 and 12 weeks after operations was examined by Micro-CT. Generally, the callus was continuously formed and mineralized over time in both groups. The callus formation and mineralization was observed mainly in periosteal region at 4 weeks while it was observed mainly in intercortical area at 8 and 12 weeks. The relative density of mineralized callus remained the same during the healing period. In ORIF group, at 4 weeks, there was less callus formation. The callus was observed within the defect region and did not bridge the gap. At 8 weeks, the process of callus formation and mineralization continued and the defect zone was bridged by newly formed bone and callus. The majority of fractures (13 of 14) obtained bone healing. At 12 weeks, the mineralized callus continued to form. By contrast, in MIPO group, the callus formation and mineralization experienced a more rapid process in early stage. The ratio of callus volume to bone volume in MIPO group was a little higher at 4 week time point than in ORIF group. The fracture area was bridged at 8 weeks in 13 out of 14 animals. At 8 and 12 weeks, the difference of the volume ratio of callus and bone in two groups was not significant. Results after 4, 8 and 12 weeks for each group are summarized in Table 2.

**Table 2:** Micro-CT analysis between MIPO and ORIF group at different time points (mean ± SD).

	4w		8w		12w	
Parameters	MIPO	ORIF	MIPO	ORIF	MIPO	ORIF
Mineralized callus volume (mm^3^)	321.04±29.18	275.04±26.71	220.44±18.07	248.86±17.81	147.54±9.78	163.47±13.59
Relative mineralized callus density	0.152±0.006	0.156±0.107	0.155±0.007	0.153±0.008	0.158±0.007	0.154±0.006
Bone volume (mm^3^)	439.71±16.07	473.68±21.62	495.21±38.63	502.93±21.42	500.47±45.41	497.39±30.97
Relative bone mineral density	0.179±0.008	0.179±0.008	0.179±0.008	0.178±0.009	0.176±0.007	0.179±0.008
Callus volume/bone volume	0.73±0.06	0.58±0.04^*^	0.45±0.03	0.49±0.03	0.29±0.03	0.33±0.03

*p< 0.05 between MIPO and ORIF.

**Fig 2:**
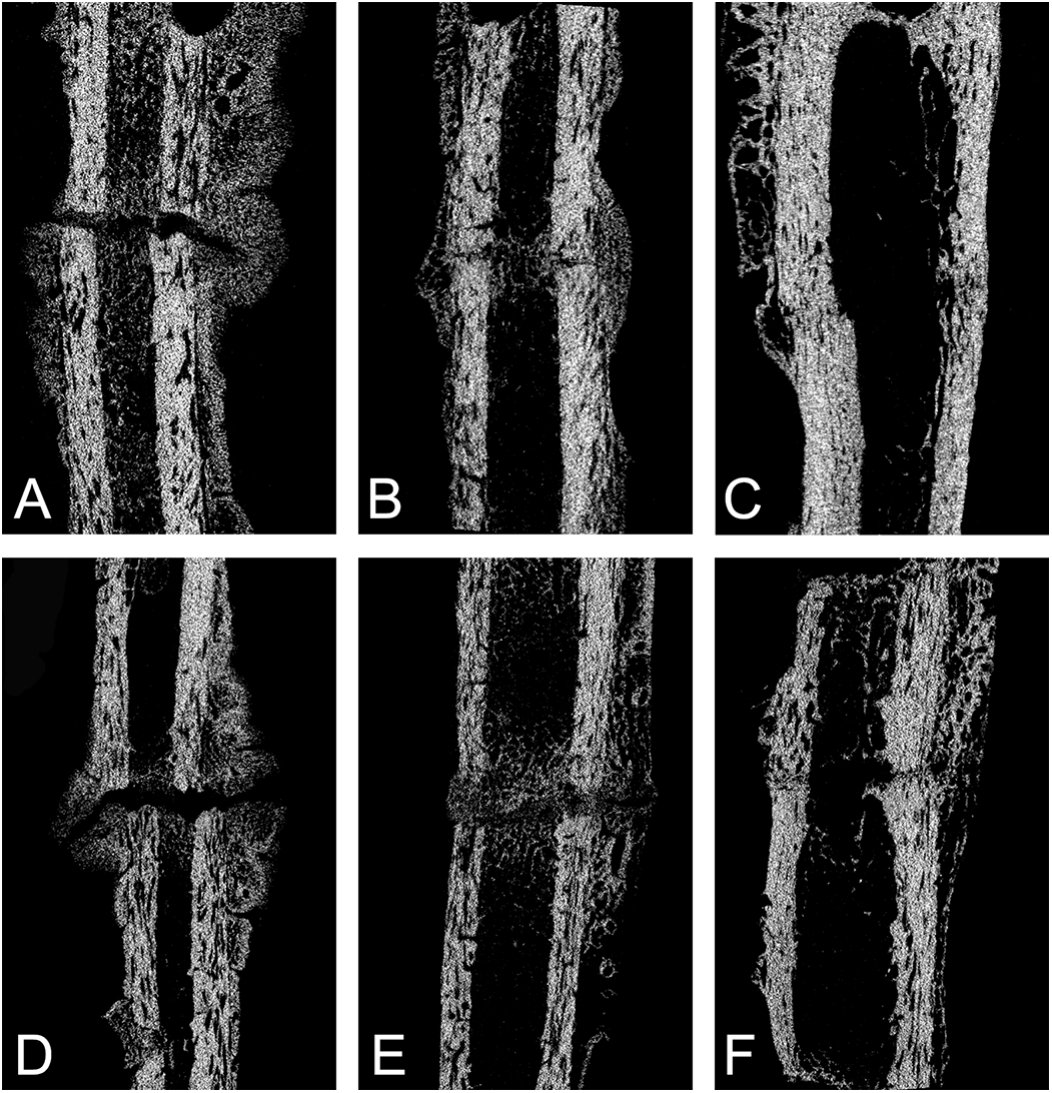
Micro-CT images of the fracture healing progression in MIPO group (A: 4 weeks; B: 8 weeks; C: 12 weeks) and ORIF group (D: 4 weeks; E: 8 weeks; F: 12 weeks).

Fig 2 provides visual verification of these tabulated results. In the MIPO group, the fracture gaps were observed to be bridged partly by more mineralized callus than that in ORIF group after 4 weeks. After 8 weeks, the fracture gaps were filled with more mineralized callus, which was also observed at 12 weeks time point. During the course of the experiment, the mineralized callus originated mainly from the periosteal regions before 4 weeks, and turned to be generated within the intercortical regions at 8 and 12 weeks in both groups.

### 3.3 Fluorochrome labeling histomorphometrical analysis

**Fig 3:**
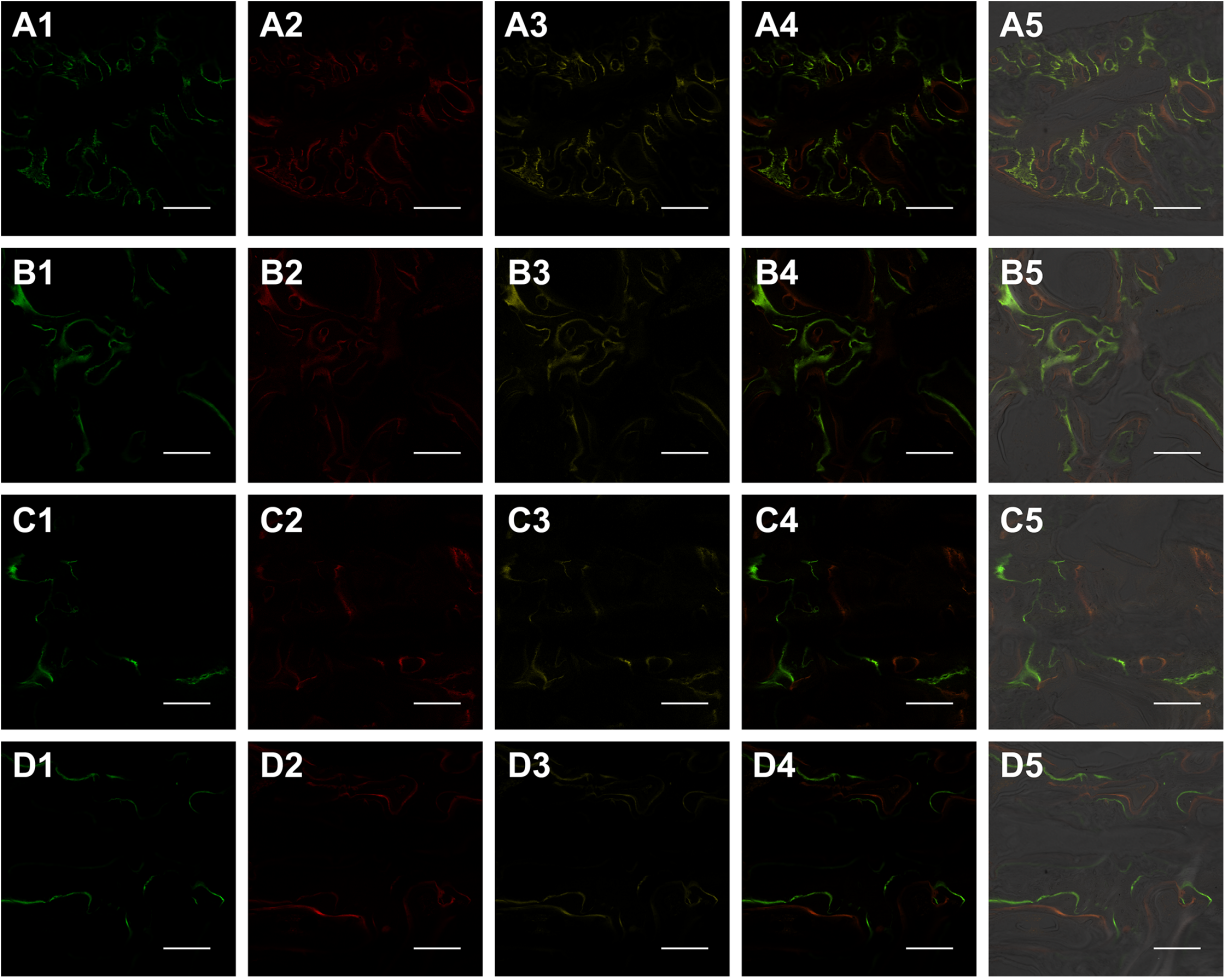
New bone formation and mineralization was monitored histomorphometrically by CA (green), AL (red) and TE (yellow) fluorescent quantification. The images of (A1-3), (B1-3), (C1-3) and (D1-3) represents the labeling at 2(A1, B1), 4(A2, B2), 6(A3, B3), 8(C1, D1), 9(C2, D2),and 10(C3, D3) weeks after operation. A, C and B, D represents confocal LASER microscope for MIPO group and ORIF group, respectively. A4, B4, C4 and D4 represent merged images of the three fluorochromes for the same group. A5, B5, C5 and D5 represent merged images of the three fluorochromes and a plain confocal laser microscope image. Scale bar = 2mm.

**Fig 4:**
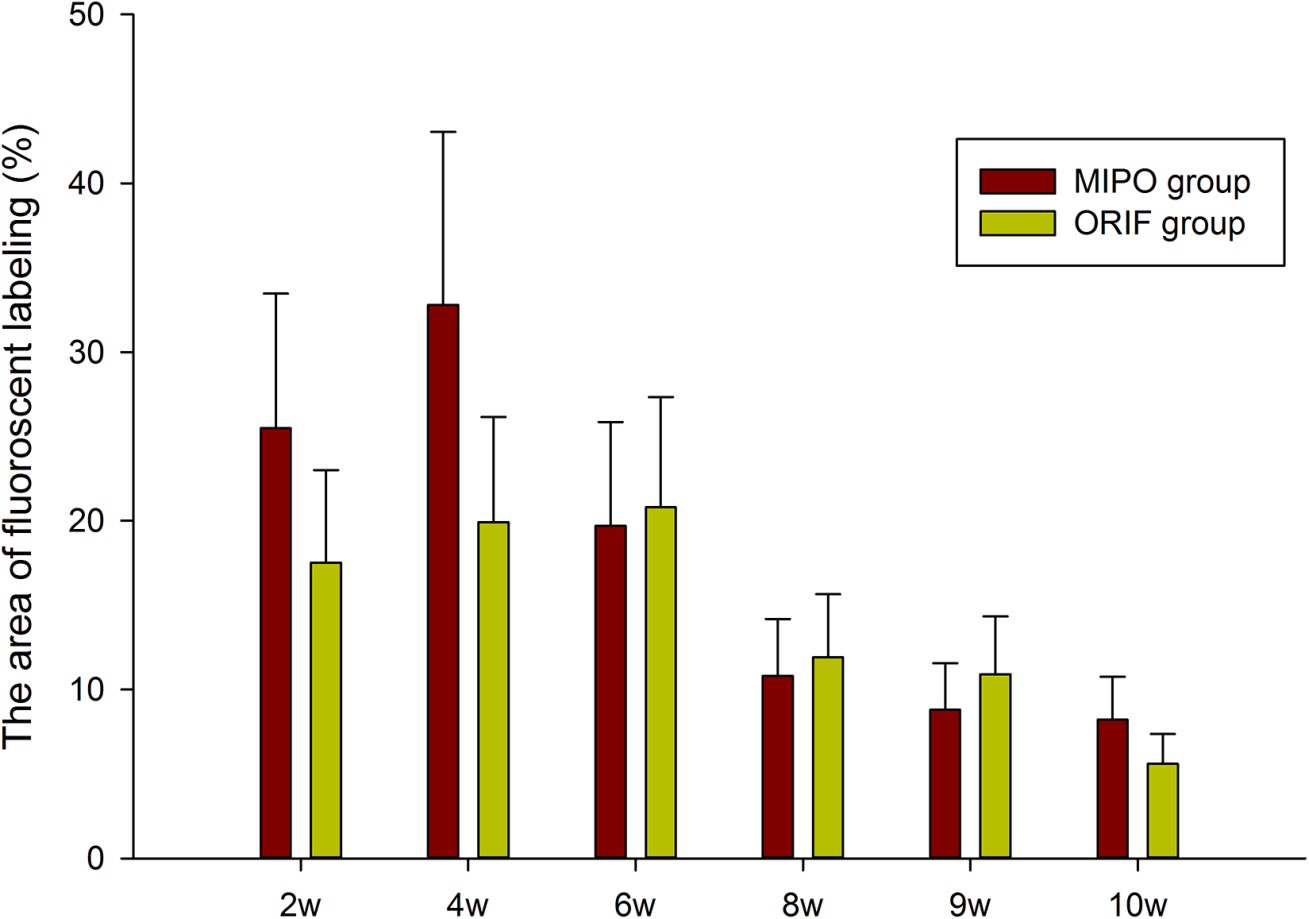
The graph demonstrates the percentage of each fluorochrome area in two groups (* indicates significant differences p < 0.05).

New bone formation and mineralization were determined histomorphometrically by caclein, alizarin red and tetracycline fluorescent quantification, which represented the level of callus mineralization at different time periods. At 2 weeks, the percentage of CA labeling (green) in dogs sacrificed at 8 weeks in MIPO group was 3.52 ± 0.23%, which was more than ORIF group at 2.84 ± 0.19% (Fig 3A1 and 3B1). There were significant differences between the two groups (*p*<0.05; Fig 4). At 4 weeks, the percentage of AL labeling (red) was 5.11 ± 0.30%, and 4.43 ± 0.27% for MIPO group and ORIF group, respectively (Fig 3A2 and 3B2). There were significant statistical differences between the two groups (*p*<0.05; Fig 4). At 6 weeks, the percentage of TE labeling (yellow) was 2.65 ± 0.15% and 2.58 ± 0.11% for MIPO group and ORIF group, respectively (Fig 3A3 and 3B3), with no significant differences between the two groups (Fig 4). From 8 weeks, the percentage of labeling decreased to a lower level in both groups (Fig 3C1–3C3 and 3D1–3D3), and there was no significant difference between the two groups (*p*>0.05; Fig 4). Taken together, these data indicate that, in MIPO group, the formation and mineralization of new bone was promoted at an earlier stage, as there was larger fluorescent area in periosteal and intercortical zone at 2 and 4 weeks post-operation than that in ORIF group. After 6 weeks, the new bone formation and mineralization turned to be similar in the two groups.

### 3.4 Histological findings

**Fig 5:**
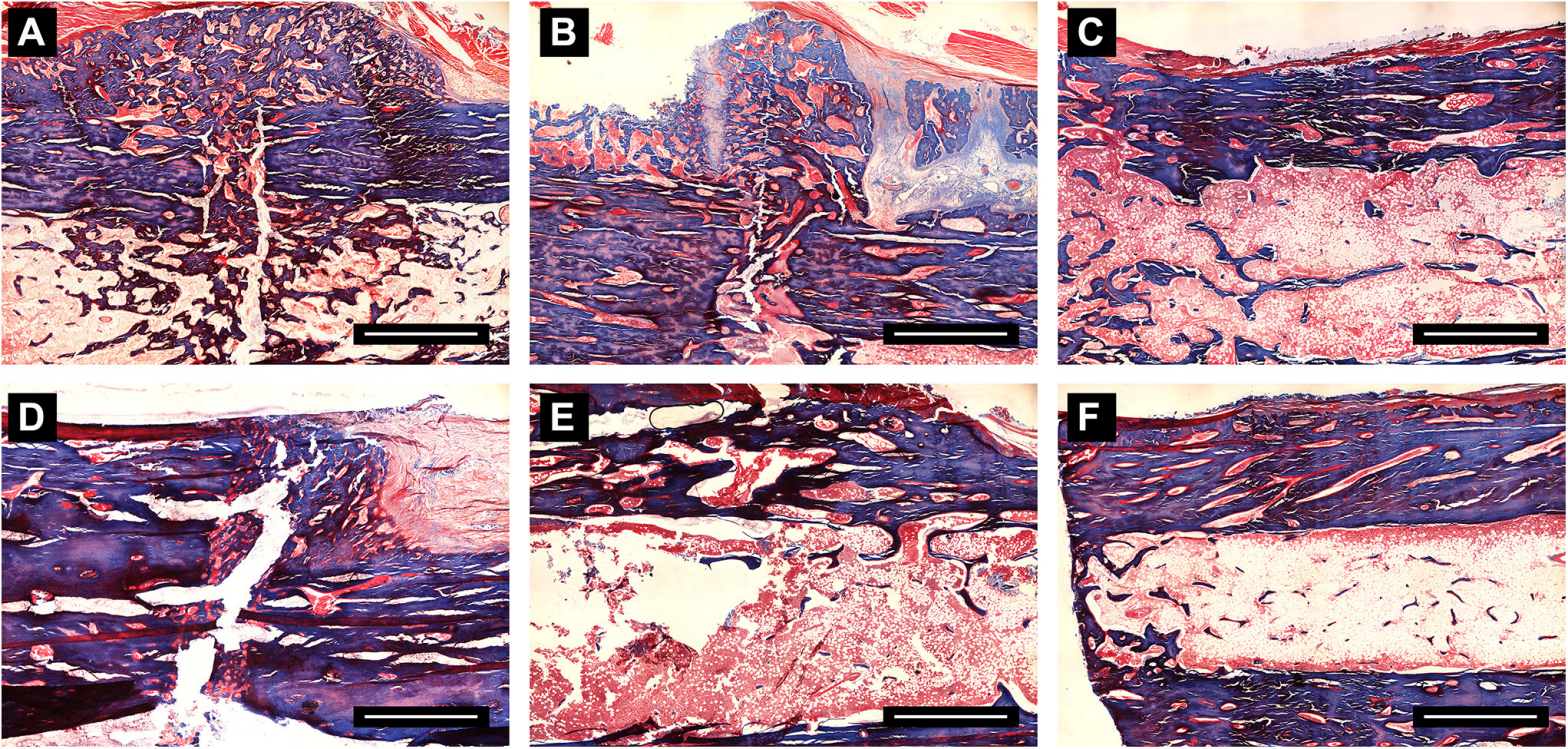
The photomicrograph of callus formation and mineralization in fracture area from specimens with Masson staining. The whole images are representative slices of two groups: 4w (A), 8w (B) and 12w (C) in MIPO Group; 4w (D), 8w (E) and 12w (F) in ORIF Group. Scale bar = 200μm.

Histological examinations were performed for two groups under light microscopy. Generally, The callus was continuously formed and mineralized over time in both groups. The newly formed bone could be observed in the periosteal and endosteal regions. At 4 weeks, more newly formed callus could be seen around the fracture site in MIPO group than that in ORIF group, while the gaps were not bridged in both groups. In terms of 8 weeks, the fracture regions were observed to be bridged with newly formed bone, as was found in the Micro-CT analysis. At 12 weeks, the fractures healed in both groups with complete bridging of the fracture gap by mineralized bone (Fig 5).

## Discussion

Open reduction and internal fixation with plate osteosynthesis is the traditional method of surgical treatment for fractures[2]. The goal of surgical treatment is anatomic reduction with reconstruction of bone length. However, there still remain some problems such as increased soft tissue stripping, infections and extensive scars. In contrast, proper alignment can be acceptable with MIPO technique as the fracture is not exposed to obtain direct reduction[19]. MIPO is an emerging technique and has been found to result in acceptable reduction and alignment for both simple and comminuted fractures[20]. The main advantage of MIPO is that it can preserve soft tissue and periosteal circulation, which are benefit to fracture healing.

To investigate the mechanism of the process of fracture healing with different surgical approach, we performed this experiment in beagles, and got the bone specimens for further measurements. The ulna was chosen as the fracture model for that the anatomical structure of canine forearm is similar with that of human, so existing internal fixation can be used for the reduction of canine ulna fracture. Moreover, the beagles bear weight mainly by their posterior limbs, so the risk for loosen and rupture of the fixation is lower when the plates were fixed in forearms.

In our study, LCP was chosen as the internal fixation device for the ulna fractures. LCP has been preferred for plate osteosynthesis of fracture as it is a kind of fairly strong implant in comparison to the strength of bone, so the early stress fracture of the implant may be prevented. The advantages of LCP include strong fixation due to the locking between plate and screws, and blood supply preservation due to the minimal contact between plate and cortical bone[21].

The X-ray images were taken for several times after operation, and the ulna samples were harvested at prescribed time for Micro-CT and histomorphometrical analysis to monitor the process of bone mineralization. These comprehensive data may be helpful to assess the outcome of MIPO technique in fracture treatments. To our knowledge, this is the first *in-vivo* report to study the mechanism of bone healing process using different plating techniques.

In people, fracture malalignment is one of the sequela of MIPO[22, 23]. In this experiment, both osteosynthesis techniques resulted in ideal frontal plane alignment. Severe rotational or frontal angulation malalignment were not observed in both groups.

A previous veterinary study comparing the bone healing between fractures treated by MIPO and ORIF indicated that dogs treated with MIPO technique healed with more callus formation[24]. In our series, all ulna fractures treated by MIPO obtained fracture union by secondary bone healing, as was suggested by the callus formation monitored at radiographic and histological observation. The callus formation and mineralization could be seen in an earlier stage in MIPO group compared to that in ORIF group. As is demonstrated by sequential fluorescent labeling and histological observation, the callus formation could be observed at 2 weeks and rose more rapidly to 4 weeks post-operation in MIPO group. By contrast, in the fractures stabilized with ORIF, callus formation was not that much in early stage as that in MIPO group. The callus formation in ORIF group also peaked at 4 weeks after operation. From 6 weeks on, the callus area seen in both groups began to reduce and did not show significant difference. The results were also supported by callus volume calculated by Micro-CT analysis.

Callus formation occurs under situations of relative stability of the implant-bone construct, and is thus considered to be the hallmark of secondary bone healing[25]. The more newly formed callus in MIPO group suggests that MIPO provides appropriate mechanical environment to stimulate the callus formation[26].

The preservation for periosteal vasculature during the osteosynthesis process in MIPO group may contribute to the rapid callus formation and mineralization. Forouk et al. performed a cadaveric study to study the effects of the two surgical plating techniques on femoral vascularity. They found that MIPO could maintain the integrity of the perforating artery and nutrient artery and contributed to superior periosteal and medullary perfusion[27]. In the operations, we found that the plate insertion tunnel was not epiperiosteal in MIPO group, while the periosteal stripping was observed in ORIF group, which was corresponding to the cadaveric study of Forouk. The extraosseous blood supply is formed by the periosteal arteries, which provide cells, oxygen, and other nutrients to the repairing site and contribute substantially over the early phase of fracture healing[28]. MIPO technique, with less periosteal stripping, may facilitate the bone healing by preserving the soft tissue and promoting oxygen tension in fracture hematoma. In contrast, the associated iatrogenic trauma for periosteum of ORIF may lead to the delay of fracture healing in initial phase[29].

In this study, the two plating techniques utilized had no significant difference on the union rate of fracture, although MIPO is a more biological-friendly procedure, which allows closed reduction without exposing the fracture area. One limitation of MIPO compared to ORIF is that the anatomic reduction may not be effectively obtained without direct vision, which may lead to larger fracture gap. Even though, there were no significant difference in final outcomes between two groups. These observations go along with other researches[30–32]. No loosening and disruption of plates and screws occurred as LCP offered satisfactory internal fixation.

The MIPO technique was straight-forward once reduction had been accomplished. In the operation, we found that the space between the ulna and extensor carpi ulnaris muscle tendon was a simple and effective hallmark for inserting the plate into distal part.

MIPO is generally considered to achieve better results in comminuted fractures[33], whereas simple fractures are better treated with open reduction[13]. In this study, the union rate was nearly equivalent and satisfactory in both groups. All simple fractures in MIPO group healed well. A prospective randomized study found that MIPO was equivalent to ORIF as a effective and safe method for humeral shaft fractures in simple types when the surgeon is experienced in MIPO technique[34]. Consequently, we assume that MIPO technique may be a useful method for fracture treatment, regardless of the fracture classification.

## Conclusion

In our experiment, the application of MIPO technique with LCP offered a satisfactory combination in terms of fracture fixation and soft tissue sparing. Operative treatment with LCP for bone shaft fractures can be used to obtain stable fixation. The advantage of MIPO for preserving periosteal blood supply may promote the early callus formation and mineralization. As the union rate of fracture treated by MIPO is comparable to conventional open reduction, we assume that the MIPO technique for bone shaft fractures should be favored, particularly when the soft tissue is in critical condition.

## Supporting Information

S1 ARRIVE Checklist (PDF)
